# VIP syndrome in surgical oncology: ethical and clinical challenges in resource-limited settings

**DOI:** 10.3389/or.2025.1683378

**Published:** 2025-10-17

**Authors:** Mukurdipi Ray, Hema Siri Kottu

**Affiliations:** Department of Surgical Oncology, B.R.A.I.R.C.H, All India Institute of Medical Sciences, New Delhi, India

**Keywords:** VIP syndrome, equitable care, resource utilisation, doctors treating doctors, ethical challenges

## Introduction

VIP (Very Important Person) Syndrome, a term coined by Dr.Walter Weintraub in 1964, describes the phenomenon where high-profile patients receive preferential treatment that deviates from standard medical protocols. At the outset of our medical careers, we all take the Hippocratic oath (1), committing to treat all individuals with equal dignity and respect. However, challenges arise in practice, particularly with patients seeking VIP treatment. These challenges often stem from management interference or external pressures that influence patient care. This issue is not limited to patients of high socioeconomic or professional status; it extends to relatives of healthcare providers who expect special treatment. Even within the medical community, when treating fellow doctors, there can be a tendency towards overcaution, leading to undue anxiety over minor conditions. Addressing these challenges requires maintaining the principles of the Hippocratic oath while navigating pressures that may compromise equitable patient care.

In a 2017 survey encompassing hospital-based physicians from eight U.S. institutions, a significant number reported feeling pressured by patients, their families, and hospital representatives to provide unnecessary care to VIP patients (2). The researchers highlighted that managing VIP patients poses challenges not only for physicians but also for the patients themselves.

A survey conducted to assess doctor-patient dynamics revealed that 11 of the 21 doctors involved reported that their physician-patients attempted to dictate their own care. The notion that “doctors make the worst patients” highlights the difficulties healthcare professionals face when treating their peers. This reinforces the importance of maintaining professional boundaries and ensuring that treatment remains patient-centered and evidence-based, regardless of the patient’s background (3).

To maintain equitable care based on medical necessity and clinical acuity, healthcare providers must ensure their workflow remains balanced. Setting clear limitations on accommodations for VIPs and establishing uniform protocols can help prevent deviations in care. Defining what is acceptable in terms of special treatment allows for consistency and fairness, ensuring that the care provided continues to prioritize the health and safety of all patients (2).

## Challenges faced by oncosurgeons with VIP patients

1. Demanding preferential Treatment: VIP patients often request special considerations such as early surgeries or preferential treatment in outpatient departments (OPDs) and wards. However, rushing surgeries without proper pre-operative optimization, such as managing recent smoking history or uncontrolled diabetes, can increase perioperative complications significantly. Incidence of obesity among people with high socioeconomic status is well known and in itself has adverse postoperative outcomes. In the current study we noted a higher BMI(Body Mass Index) among private admissions compared to general wards.
2. Special care demands in wards: VIP patients may expect more frequent visits from their treating surgeons, which can strain resources in busy healthcare settings like government institutions in India. This extra time spent on one patient could otherwise benefit other patients in need. The desire for prolonged intensive care unit (ICU) stay for “extra attention” can reflect a combination of anxiety, fear of complications, or perhaps a perception that her needs are not being met adequately in a standard ward setting. This situation can lead to conflicts in care priorities; while the staff aims for efficient use of resources and optimal discharge protocols, the patient’s request for longer stay can create complications. A 65 year old patient, who was mother of a fellow doctor working at the hospital, had developed hospital acquired pneumonia during her ICU stay, which the doctor persisted on prolonging despite the condition of the patient. They would frequently intervene in postoperative medications, discontinuing intravenous fluids on their own initiative, and requesting prolonged antibiotics without necessity. Mobilization was often delayed despite repeated assurances, due to an excessive fear of complications. This overcautious behaviour inturn led to more complications in a case which was otherwise not a major surgery
3. Insistence on unnecessary investigations: There are instances where VIP patients or their relatives push for unnecessary tests. For example, a 60-year-old female with early breast cancer presented to us, her relatives insisted on a PET CT (Positron Emission Tomography–Computed Tomography) scan, despite risks like false results in regions with high tuberculosis prevalence. Her PET CT showed increased uptake in mediastinal lymphnodes, and she had to undergo endobronchial FNAC(Fine needle aspiration cytology) for adequate staging, which only turned out to be reactive. Delays caused by such demands can lead to disease progression and worsened outcomes (4).
4. Administrative pressures: Higher officials and administrators sometimes exert pressure on doctors to provide special attention to certain patients, irrespective of the already demanding workload. This administrative interference can create stress and affect patient care (5).
5. Reliance on unverified online information: With access to abundant medical information online, some patients arrive with preconceived diagnoses and treatment plans, often from unreliable sources. This can lead to distrust in medical professionals and challenges in establishing effective doctor-patient communication. Google can only provide information not knowledge, but the patient considers it as knowledge (6,7).
6. Seeking multiple consultations across physicians: Consulting multiple doctors in the same hospital, can lead to lack of trust among doctors. In the field of medicine, no faith–no gain is the norm (8).
7. Requesting for personal contact of the doctors and calling them for minor ailments.
8. Miscellaneous: A young male who underwent hemicolectomy, he would not shower despite persistence on hygiene, taking advice from relatives. He developed surgical site infection 6 days postoperatively, and minimal drain site discharge. He had unexplained pain abdomen which would not resolve on oral medications. Unexplained palpitations and respiratory distress, although the monitored vitals were always normal. CT abdomen was performed to rule out any leak, which only turned out to be normal. He was then counselled by a psychotherapist and later improved.

## Challenges faced by patients

1. Seeking multiple opinions and unnecessary investigations: Patients often seek multiple opinions from different doctors and healthcare facilities, which can lead to confusion and unnecessary delays in treatment. This practice also results in exposure patients to additional risks such as radiation without added benefit.
2. Family involvement and emotional support: In India, hospitalized patients are typically accompanied by family members who take extended leave to provide support. This familial presence boosts patients’ self-esteem, facilitates communication with healthcare professionals, and enhances overall satisfaction with care (9). However, hiring external help to assist patients, while practical, may not offer the same emotional support and involvement as family members. In such cases, hired help may lack the understanding and commitment to support post-operative care effectively, potentially hindering patient’s rehabilitation.
3. Early admissions and surgical interventions without optimization: Some patients, due to various reasons including influence from higher authorities, may be admitted early or undergo surgeries without proper optimization. Although preoperative incentive spirometry is not routinely recommended to reduce postoperative pulmonary complications, several systematic reviews suggest there is some low-quality evidence supporting its effectiveness. Early mobility and ambulation are generally recommended to promote airway clearance and reduce pulmonary complications. For instance, consider the case of a 58-year-old female with advanced ovarian cancer. Due to external pressures from management, she was admitted and operated on without adequate preoperative preparation. These pressures extended beyond admission, influencing decisions such as prolonged ICU stays postoperatively, which are not necessary. Consequently, her recovery was complicated by delayed ambulation, leading to postoperative basal atelectasis and subsequent pulmonary complications. Her hospital stay extended to 12 days, whereas patients typically undergoing similar surgeries are discharged within 5 days.
4. Hiding the history: Missing or inaccurate information could lead to misdiagnosis of conditions, unnecessary tests or procedures, inappropriate or ineffective treatments, delays in proper care. A 40 year old patient, who was well educated, diagnosed with ovarian cancer. She is a known carrier of beta thalassemia trait, she withheld the information during the course of her stay. She had postoperative bleeding and was reexplored because of anastomotic leak.
5. Impact on non-VIP patients The resources and attention devoted to VIP patients can indirectly affect non-VIP patients. When physicians and staff are preoccupied with high-profile cases, the care and attention given to regular patients may diminish, leading to delays or substandard treatment (10).

## Challenges faced by hospital staff

1. Frequent and disruptive visitors: Hospital staff often deal with frequent and untimely visits from patients’ relatives, disregarding security and hospital protocols. This disrupts unit operations and increases the risk of infections, particularly among immunocompromised patients like those in ICU. Giving precedence to the insights of previously hospitalized relatives over medical professionals’ recommendations is one problem frequently encountered in VIP patients.
2. Prolonged hospitalization requests: Surgeons may encounter requests for unnecessary prolonged hospital stays from patients or their caregivers, especially in private ward admissions. This extends the disease-specific mean duration of hospitalization beyond what is medically necessary. Repeated requests for readmission for minor issues and filing complaints can indicate a strong desire for attention or reassurance. Three patients of breast surgery could get operated in the same time frame as one patient of breast surgery in a private ward admission with extended stay.
3. Demanding extra care and superiority complex: Patients or their relatives sometimes perceive themselves as superior and demand exceptional care. For example, a 56 year old, close relative of a judicial executive was admitted for carcinoma colon and underwent right hemicolectomy, postoperative course was unremarkable, and her drain was removed by a first year surgical resident with strict protocol adherence, the following day the department head received a mail from director office asking for an explanation for causing pain during the procedure. Patients with connections to the medical field (like the family of a medical resident) may have heightened expectations regarding their treatment. They expect the drain to be removed by department head based on their status. Such situations can lead to stress for the medical residents and staff involved, as they navigate the complexities of providing care while facing potential scrutiny or backlash.
4. Overcautiousness towards relatives: Surgeons themselves may exhibit overcautious behaviour when treating their own relatives, which can lead to excessive interventions or prolonged hospital stays. During the treatment of a close relative of a medical professional, we often encounter requests to extend the duration of antibiotic courses or to discontinue intravenous fluids based on personal comfort rather than medical necessity (11).
5. Extended duration of hospital stay: At our oncology center, the mean duration of hospital stays in private ward admissions is notably longer compared to general ward admissions, significantly impacting hospital resources and patient turnover.

## Caring of VIP patients: do’s and don’ts

The five principles that we propose based on our experience in treating these patients are:

1. Establish stringent admission criteria: Implementing strict admission criteria and adhering to a checklist can reduce management interference in patient care decisions.
2. Enhance communication: Effective communication is crucial in managing expectations and addressing concerns. It may be beneficial to ensure that the patient feels heard and understood while politely explaining the rationale behind treatment decisions.
3. Equitable distribution of care: Ensuring that time and care are allocated based on patient needs rather than their status or demands promotes fairness and efficiency in healthcare delivery.
4. Support for security staff: Providing adequate support to security personnel to enforce visitation rules helps maintain unit cohesion and efficient patient management, minimizing disruptions from excessive visitors.
5. Enforce strict discharge criteria: Implementing clear guidelines for hospital and ICU admissions and discharges, and informing patients preoperatively about the importance of early ambulation for faster recovery.

## Conclusion

We have observed in our practice, among the few people who develop faith in doctors and adhered to them all along have shown better results and discharged on time. While special accommodations for high-profile individuals may be acceptable if they do not compromise access or the quality of care for others, healthcare providers must be vigilant. An important aspect of managing VIP syndrome is the role of disclosure itself. The act of sharing real-world clinical experiences, whether through academic publications or institutional forums, can serve as a powerful strategy to raise awareness of this issue. Documenting and disseminating such cases sensitises healthcare professionals to the ethical and clinical challenges involved, stimulates dialogue within institutions, and encourages the development of uniform policies to safeguard equity in patient care. By openly acknowledging these situations, we not only validate the experiences of clinicians but also promote preventive strategies that can mitigate the negative consequences of preferential treatment.
